# Unseen Dangers—The Role of Invasive Species in the Spread of Zoonotic Diseases in Europe

**DOI:** 10.3390/pathogens14020159

**Published:** 2025-02-07

**Authors:** Myriam Kratou, Alejandro Cabezas-Cruz

**Affiliations:** 1 Laboratory of Microbiology, National School of Veterinary Medicine of Sidi Thabet, University of Manouba, Manouba 2010, Tunisia; 2 ANSES, INRAE, Ecole Nationale Vétérinaire d’Alfort, UMR BIPAR, Laboratoire de Santé Animale, 94700 Maisons-Alfort, France

In a recent study, Klink et al. [1] investigated the potential of raccoon dogs (*Nyctereutes procyonoides*) and raccoons (*Procyon lotor*) as reservoirs and vectors for vector-borne and zoonotic pathogens in Schleswig-Holstein, Germany. These invasive species, native to East Asia and North America, respectively, have adapted to European ecosystems [2], raising concerns for human and animal health. The study screened these animals for pathogens such as *Leptospira*, *Rickettsia*, and *Borreliella* spp., with findings that shed light on their capacity to harbor and potentially spread infectious agents.

Raccoon dogs and raccoons exhibit key traits such as high adaptability [3,4], omnivorous diets [5], and rapid reproduction that contribute to their success as invasive species in Europe [6]. These characteristics disrupt local biodiversity and facilitate interactions with native wildlife and domestic animals [7,8], potentially increasing the risk of disease transmission [9]. While both species show similarities in pathogen infection patterns, raccoon dogs tend to inhabit natural environments, including forests, wetlands, and rural areas, avoiding densely populated urban centers [10,11]. This preference for non-urban areas reduces direct human contact, but increases interactions with wildlife reservoirs of pathogens [4]. These animals often share dens with species such as badgers and foxes [12], increasing their exposure to ectoparasites like ticks, vectors of pathogens like *Borreliella* spp. that cause Lyme disease [13] and *Rickettsia* spp., which leads to rickettsiosis [14,15,16]. Their omnivorous diet, which includes small mammals, amphibians, insects, and plants, also raises their likelihood of encountering pathogens like *Leptospira* spp. and other rodent-borne diseases [5,12]. On the other hand, raccoons are highly adaptable, and commonly inhabit urban and suburban areas, bringing them into closer contact with humans and domestic animals compared to raccoon dogs [17]. This proximity elevates the risk of zoonotic disease transmission [1]. While raccoons frequently forage in areas with high tick densities, their interactions with large wildlife are relatively limited. Moreover, as omnivores, raccoons exhibit opportunistic feeding behaviors, often scavenging in garbage and human food waste [18,19]. This feeding strategy increases their exposure to pathogens associated with human environments, such as *Leptospira* spp. and *Salmonella* [20,21].

The study by Klink et al. [1] highlighted differences in the roles of raccoon dogs and raccoons as carriers of *Leptospira* spp. and tick-borne pathogens. Their study found *Leptospira* spp., a bacterial pathogen responsible for severe leptospirosis in humans [22], in approximately 19% of raccoon dogs and 7% of raccoons, underscoring the need for monitoring these populations for zoonotic threats [1]. Raccoon dogs exhibited a nearly threefold higher infection rate than raccoons, which may be linked to their dietary habits, involving more predation on small mammals, increasing their exposure to rodent-borne pathogens such as hantaviruses, *Toxoplasma gondii*, and *Leptospira* spp [23,24]. Additionally, the greater susceptibility of juvenile raccoon dogs to *Leptospira* suggests that these animals may act as reservoirs, shedding the bacteria into the environment, potentially contaminating soil and water, which can spread the infection to humans, livestock, and other wildlife [25,26].

In their examination of tick-borne pathogens, Klink et al. [1] found a notably low prevalence of *Borreliella afzelii*, a key agent of Lyme disease in Europe, and no infections of *Rickettsia* spp. in either raccoon dogs or raccoons. This low detection rate is intriguing, especially considering that only one individual from each species tested positive for *Borreliella* spp., and no *Rickettsia* infections were found. Such findings may suggest a limited role for these animals in the ecology of these pathogens, at least within the sampled population in Schleswig-Holstein. However, the ecological implications are profound, given the observed behavioral overlap where both species share dens with native predators such as red foxes and badgers [12]. This interspecies interaction could potentially enhance the exchange of ectoparasites, including ticks, thereby facilitating the broader transmission dynamics of tick-borne diseases across different wildlife populations.

As Klink et al. [1] emphasized, long-term health monitoring of raccoon dogs and raccoons is essential for understanding and managing their roles in zoonotic disease transmission. These invasive species play complex roles in host–pathogen ecosystems, necessitating detailed investigations to unravel observed nonlinearities and to develop adaptive management strategies that address each species’ specific contributions to pathogen ecology [27]. In order to maximize the impact of these strategies, future studies should investigate the effects of co-infection and combined infestations on raccoon dogs and raccoons. As shown by Klink et al. [1], these species were primarily infected with ticks, but also harbored fleas, lice, and louse flies, which can carry a range of pathogens [28,29,30]. Concurrent infestations with these insects increase the risk of multiple pathogen carriage, leading to co-infections [31,32]. For example, *Yersinia pestis*, the causative agent of plague, primarily transmitted by fleas, can result in bubonic, septicemic, or pneumonic plague [33]. These co-infections may increase the reservoir competence of raccoon dogs and raccoons for multiple pathogens, promoting pathogen transmission within and between species, and elevating the risk of outbreaks impacting wildlife, domestic animals, and humans [34]. Furthermore, ectoparasites may detach from their hosts post-mortem, especially during transport and handling, potentially leading to underestimation of infestation rates [35]. This detachment can distort data on parasite loads, obscure pathogen exposures, and complicate the understanding of vector–host relationships. Detached ectoparasites may still harbor significant pathogen loads, contributing to co-infection scenarios or independent transmission cycles, reducing opportunities to detect and study vector-borne pathogens [35]. Advanced molecular techniques, such as next-generation sequencing (NGS), can be employed to identify the full spectrum of pathogens in collected samples, including emerging or underexplored pathogens [36]. These methods can uncover hidden or co-occurring infections and provide a comprehensive understanding of the microbiota in raccoon dogs and raccoons, offering valuable insights into a profile of pathogen interactions [37].

In summary, Klink et al. [1] highlight the complex challenges posed by the invasive species raccoon dogs and raccoons in host–pathogen ecosystems. Both species play distinct, yet interconnected, roles in pathogen ecology, as their association with ticks contributes to local tick populations and the potential transmission of Lyme disease and other tick- and rodent-borne pathogens. Advancing our understanding of how raccoon dogs and raccoons influence the epidemiology of *Leptospira* and tick-borne diseases through research and collaboration will enable better anticipation and mitigation of the risks these species pose to humans, animals, and ecosystems.
